# Fluorine-Lean Phosphonated Polymers of Intrinsic Microporosity with High Oxygen Permeability as a PEMFC Catalyst Layer Ionomer

**DOI:** 10.1021/acsaem.5c00265

**Published:** 2025-03-28

**Authors:** Theresa Stigler, Tamas Nemeth, Patrick Fortin, Simon Thiele, Jochen Kerres

**Affiliations:** † Forschungszentrum Jülich GmbH, Helmholtz Institute Erlangen-Nürnberg for Renewable Energy (IET-2), Cauerstr. 1, Erlangen 91058, Germany; ‡ Department of Chemical and Biological Engineering, Friedrich-Alexander-Universität Erlangen-Nürnberg, Immerwahrstr. 2a, Erlangen 91058, Germany; § Department of Sustainable Energy Technology, SINTEF Industry, Trondheim 7034 Norway; ∥ Chemical Resource Beneficiation Faculty of Natural Sciences, North-West University, Potchefstroom 2520, South Africa

**Keywords:** polymer of intrinsic microporosity, fluorine-lean, ionomer, catalyst layer, oxygen diffusion coefficient, PEMFC

## Abstract

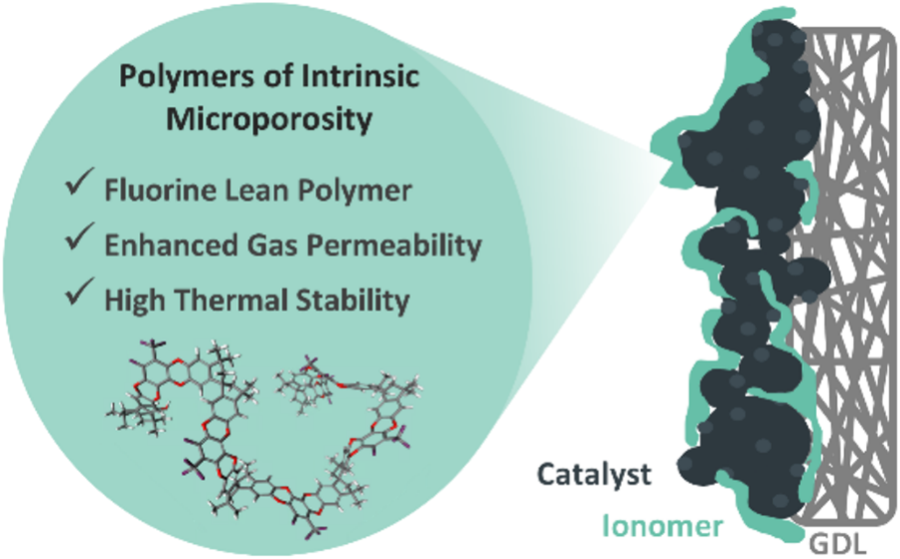

An essential component of proton exchange membrane fuel cell (PEMFC) technology is the catalyst layer ionomer, serving as the binder and transport matrix responsible for the macroporous electrode structure and the regulation of proton and reactant gas supply to the catalyst interface. To improve the mass transport properties of the catalyst layer, we developed a fluorine-lean phosphonated polymer of intrinsic microporosity (pPIM). The highly kinked structure of the pPIM results in an ionomeric network with increased porosity to promote enhanced gas diffusion through the ionomer layer, while the incorporation of phosphonic acid head groups provides efficient proton conduction. Increased gas permeability of the ionomer is an important factor for effectively mitigating local transport losses that typically occur at high current densities. In situ PEMFC tests were carried out where the pPIM was utilized as the ionomer in the catalyst layer on both the anode and the cathode side. The ionomer-to-carbon (I/C) ratio was varied to evaluate its impact on the oxygen diffusion coefficient and overall fuel cell performance. A higher oxygen diffusion coefficient was achieved with the pPIM using an I/C ratio of 0.2, compared to the Nafion-based catalyst layer.

## 1 Introduction

Fuel cell technologies employing proton exchange membranes (PEMs) are regarded as pivotal technologies for reducing anthropogenic carbon emissions. A critical component of the proton exchange membrane fuel cell (PEMFC) is the membrane electrode assembly (MEA), which comprises a PEM, one catalyst layer (CL), a gas diffusion layer (GDL) on the anode side, and one CL and one GDL on the cathode side. The ionomer within the catalyst layer plays a vital role as it strongly influences morphology and the efficiency of the catalyst layer in the electrode.^1,2^ The ionomer serves as a bridge between the catalyst surface and proton exchange membranes, facilitating the transport of protons from the membrane and reactant gases from the GDL to the catalyst surface.^3−5^ This region where the catalyst, ionomer, and gas phase meet is termed the triple-phase region. To circumvent mass transport losses during operation and ensure optimum performance of the PEMFC, it is crucial for ionomers used in the catalyst layer to be gas-permeable, allowing gaseous fuels to quickly reach the reaction sites at the catalyst.^6^ The main focus of catalyst layer optimization of PEMFC research has been on the cathode side due to the sluggish kinetics of the oxygen reduction reaction (ORR).^7^ The ORR is a three-phase heterogeneous process requiring the coexistence of O_2_ in the gas phase, electrons in the solid phase, and protons in the ionomer phase at the reaction sites of the catalyst. The study of Tabe et al.^8^ revealed that oxygen transport resistance through the ionomer coating on the Pt/C influences the rate-determining processes of the cathode CL activity under normal operating fuel cell conditions. Nonoyma et al.^9^ stated that the total oxygen transport resistance comprises three components: molecular diffusion, Knudsen diffusion, and permeation through the ionomer film, with oxygen permeation being the predominant contributor.^9^ Consequently, recent research has focused on improving the oxygen permeability of the ionomer. Katzenberg et al.^6^ achieved enhanced gas transport with an ionomer incorporating a glassy amorphous matrix based on a perfluoro(2-methylene-4-methyl-1,3-dioxolane) backbone and Zhang et al.^10^ demonstrated improved oxygen permeation by implementing ionic covalent organic framework nanosheets into Nafion ionomer. In general, oxygen permeability can be deconvoluted into solubility and diffusion.^11−13^ Several literature reports indicate that in Nafion-based structures, oxygen solubility increases with the hydrophobicity of the polymer, whereas oxygen diffusion is influenced by the polymer’s water content. Consequently, the hydrophilic phase, which includes −SO_3_H or −PO_3_H_2_ groups and water, plays a crucial role in oxygen diffusion.^14−16^ Zhang et al.^13^ investigated the gas permeability properties of two hydrocarbon-based polymers, sulfonated poly(arylene ether sulfone) (SPES) and sulfonated poly(phenylene sulfide sulfone) (SPSS). They found that the oxygen diffusion in both polymers was influenced by volume fraction of the hydrophilic domains. A series of SPES and SPSS polymers with varying ion exchange capacities (IEC) were synthesized, and it was demonstrated that oxygen diffusion increases with increasing IEC as a result of the higher water content and larger volume fraction of hydrophilic domains associated with higher IECs. Conversely, the relative volume of hydrophobic domains made up of the hydrocarbon polymer backbone was reduced with increasing IEC, resulting in lower oxygen solubility.^13^ This inverse relationship between diffusion and solubility makes it difficult to predict the overall oxygen permeability trend. The authors noted that the overall transport properties depend markedly on the subtle variations in polymer composition and structure, with the polymer’s microstructure playing a crucial role. Well-connected channels between ionic domains are required to create efficient transport pathways for rapid oxygen diffusion.^13,17^ In recent years, the challenge of optimizing gas diffusion while preserving proton conductivity in the catalyst layer has been addressed by employing polymers with intrinsic microporosity (PIMs) as ionomer binders.^18−24^ PIMs are characterized by their high gas permeability properties, owing to the significant amount of free volume between polymer chains imparted by their rigid and contorted molecular structure that prevents efficient chain packing.^25,26^ Herein, we present a phosphonated PIM comprising a rigid, hydrophobic backbone and ion-conductive −PO_3_H_2_ groups. The physical properties of the pPIM were characterized by ex-situ methods, e.g., NMR, GPC, BET, TGA, and water uptake, while the performance of the pPIM as a catalyst layer ionomer was investigated through rotating disk electrode (RDE) measurements and in situ fuel cell tests to determine the electrochemically active surface area (ECSA) and oxygen diffusion coefficient (*D*_O2_) of the resulting catalyst layers. The I/C ratio was systematically varied to understand the influence of ionomer content toward an optimal balance of ionic conductivity and gas permeability in an operational fuel cell.

## 2 Experimental Section

### 2.1 Materials

5,5′,6,6′-Tetrahydroxy-3,3,3′,3′-tetramethyl-1,1′-spirobiindane (Spiro) and 1,2,3,4,5-pentafluoro-6-(trifluoromethyl)benzene (OFT) were purchased from BLD Pharmatech GmbH. Sigma-Aldrich was the supplier for potassium carbonate (K_2_CO_3_), sodium chloride (NaCl), sodium hydroxide (NaOH), hydrochloric acid (HCl), perchloric acid solution (HClO_4_), sulfuric acid (H_2_SO_4_), and all used solvents. Tris(trimethylsilyl)phosphite (TSP) was purchased from Manchester Organics. Spiro was dissolved in methanol and precipitated in water. All other chemicals were used as received. The Nafion membrane (N211) and the gas diffusion layers (Sigracet 22 BB – 48 × 40 cm) were ordered from Fuel Cell Store. The Pt/C catalyst (60 wt % platinum on high surface area advanced carbon support) was purchased from Thermo Scientific, and the Nafion dispersions (D521 and D2021) were purchased from Ion Power.

### 2.2 Synthesis of Polymer of Intrinsic Microporosity (PIM)

Spiro (1.00 equiv, 7.03 g, 20.20 mmol) and OFT (1.00 equiv, 4.98 g, 20.20 mmol) were dissolved in anhydrous dimethylacetamide (DMAc, 40 mL) and anhydrous toluene (20 mL) under an argon atmosphere in a round-bottom flask equipped with a Dean–Stark apparatus. Anhydrous K_2_CO_3_ (3.00 equiv, 8.46 g, 60.60 mol) was added and the reaction mixture was heated to 160 °C. After 62 min at 160 °C, the reaction mixture was cooled down for 5 min and precipitated in methanol. The polymer was filtered, dissolved, and reprecipitated in a methanol/water mixture (1:1, v:v). The filtered product was boiled in water and washed in water until a neutral pH was obtained. After drying at 70 °C overnight, a white solid was obtained (yield: 96%).^1^H NMR (500 MHz, CDCl_3_, δ [ppm]): 6.70 (s, 1H); 6.34 (s, 1H); 2.28 (m, 1H); 2.14 (m, 1H); 1.28 (m, 6H). ^19^F NMR (470 MHz, CDCl_3_, δ [ppm]): −55.19; −143.56.

### 2.3 Synthesis of Phosphonated Polymer of Intrinsic Microporosity (pPIM)

PIM (1.98 g, 3.95 mmol) and TSP (8.68 equiv, 8.00 mL, 34.30 mmol) were added in a round-bottom flask equipped with a reflux condenser under argon atmosphere. The reaction mixture was vigorously stirred for 24 h at 170 °C. The orange reaction solution was cooled and poured into deionized water. Afterward, the water was heated to 100 °C for 1 h. The formed byproduct, hexamethyldisiloxane, was separated as an oily phase. The orange dispersion was dialyzed (dialyzing tubes MWCO 6000–8000 g mol^–1^, regenerated cellulose from Spectra/Por) for 2 days by exchanging the water frequently. After the water was removed by a rotary evaporator, the dried solid was conditioned in 10 wt % H_2_SO_4_ at 70 °C for 2 h and washed with water until the pH was neutral. Drying at 85 °C afforded an orange-yellowish solid (yield: 82%). By the integral ratio between the resonances corresponding to the −CF_3_ (−54.04 ppm) and the neighboring meta fluorine atom (−145.20 ppm) in the ^19^F NMR spectrum, the phosphonation degree was determined. ^1^H NMR (500 MHz, DMSO-*d*_6_, δ [ppm]): 8.19 (m, −PO_3_H_2_); 6.88 (m, 1H); 6.28 (m, 1H); 2.25 (m, 1H); 2.08 (m, 1H); 1.23 (m, 6H). ^19^F NMR (470 MHz, DMSO-*d*_6_, δ (ppm)): −54.05; −145.20. ^31^P NMR (202 MHz, DMSO-*d*_6_, δ [ppm]): 2.41.

### 2.4 Polymer Characterization

#### 2.4.1 Gel Permeation Chromatography (GPC)

GPC measurements were employed using a SECcurity2 1260 instrument from PSS. A PSS SDV LUX GUARD was used as a guard column, and three separation columns (2x PSS SDV LUX 3 μm 1000 Å and 1x PSS SDV LUX 3 μm 10,000 Å) were applied for sample analysis. The eluent was tetrahydrofuran with a flow rate of 1.0 mL min^–1^ at 35 °C. A dual variable wavelength UV–vis (P/N 404–2107, PSS) and a refractive index detector (P/N 404–2106, PSS) were used as the detectors. The relative molecular weight was determined by calibration with narrowly distributed polystyrene standards from PSS.

#### 2.4.2 Thermogravimetric Analysis (TGA)

The thermal stability of the synthesized polymer was investigated using the TGA8000 from PerkinElmer. A heating rate of 10 °C min^–1^, a gas flow rate of 30 mL min^–1^, and synthetic air (80% N_2_, 20% O_2_) as gas environment were used for all measurements over a temperature range of 30–800 °C.

#### 2.4.3 Fourier Transform Infrared Spectroscopy (FT-IR)

FT-IR spectroscopy was performed using a 3 FT-IR spectrometer from PerkinElmer equipped with the ATR unit at a wavenumber from 4000 to 650 cm^–1^.

#### 2.4.4 Differential Scanning Calorimetry (DSC)

Differential scanning calorimetry was measured using a DSC 3+ from Mettler Toledo with a heating rate of 10 °C min^–1^ under a nitrogen atmosphere and a flow rate of 50 mL min^–1^ in a temperature range from 50 to 280 °C. All samples were measured in two cycles, whereas the first cycle was excluded.

#### 2.4.5 Ion Exchange Capacity (IEC)

Ion exchange capacities (IEC_direct_ and IEC_total_) of the polymers were determined by the salt splitting titration method at room temperature using an OMNIS-titrator of Metrohm. The solid polymers were immersed in a saturated NaCl solution for 24 h at 85 °C. The exchanged H^+^ ions were then titrated with 0.1 M NaOH to the equivalent point (IEC_direct_), and a defined excess of NaOH was added. Afterward, the solution was stored for 24 h at 85 °C to be certain that all protons of the phosphonic acid groups were deprotonated. This solution was back-titrated with 0.1 M HCl, and the IEC_total_ was calculated. IEC values are obtained from the titration results using the formula:
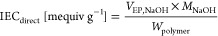

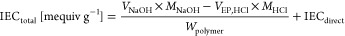
where *V*_EP,NaOH_ is the volume in mL of NaOH at the equivalent point, *V*_NaOH_ is the total volume of added NaOH, *M*_NaOH_ is the molecular weight of NaOH, *V*_EP,HCl_ is the volume of HCl at the equivalent point, *M*_HCl_ is the molecular weight of HCl, and *W*_polymer_ is the weight of the dried polymer in the acidic form. The titration was performed three times, taking the average values for IEC_direct_ and IEC_total_.

#### 2.4.6 Water Uptake (WU)

Due to its low mechanical properties, only small membrane pieces of the pPIM polymer were weighed in their dry state (*W*_dry_) in a centrifuge tube. A D2021 dispersion was used to create ∼30 μm thick reference membranes . The pPIM membrane pieces and the D2021 membranes were left for one, two, four, and 6 days at 85 °C in deionized water. The mass of the wet membrane samples (*W*_wet_) was measured after centrifugation for 20 min at 1000 rpm and subsequent separation of the water from the membrane samples. The WU was calculated from the difference between the wet and the dry mass of the membrane samples:
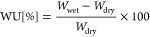


Three samples were measured for each membrane type, taking the average for the water uptake. The overall higher water uptake might stem from the measurement of small membrane fragments.

#### 2.4.7 N_2_–Sorption

The surface area of the synthesized polymer pPIM was determined via nitrogen physisorption at liquid nitrogen temperature using a Quadrasorb SI analyzer (Quantachrome Instruments) and analyzed with the Brunauer–Emmett–Teller (BET) method^27^ in a relative pressure range between 0.04 and 1 p/p°. Prior to all measurements, the samples were degassed for 24 h at 150 °C and a pressure of 0.01 mbar. The error was calculated as the standard deviation of the mean, which was determined from three measurements.

### 2.5 Characterization as Binder Material

#### 2.5.1 Ink Preparation

A prescreening of different solvents used in the ink formulation was done by examining the ECSA via rotating disc electrode measurements (see Table S2). Herein, the optimum solvent was found to be 40 wt % H_2_O in 1-propanol when using D521 as binder and 15 wt % H_2_O in dimethylacetamide (DMAc) when using pPIM as binder. The ink solutions were fabricated by adding the solvent to the dry Pt/C 60 wt % and sonicating it for 15 min. Afterward, the ionomer solution (D521 dispersion or 5 wt % pPIM dissolved in 15 wt % H_2_O/DMAc solution) was dropwise added and sonicated again for 15 min. The total solid-to-solvent content was always set to 0.25 wt %. The catalyst inks were stirred overnight. Before using the inks, they were homogenized using an ultrasonic horn (Hielscher) at an amplitude of 20% with 0.5 s for 5 min of pulsing in between at 0 °C.

#### 2.5.2 Rotating Disk Electrode Measurements (RDE)

The working electrode, a glassy carbon electrode supported by a PTFE-body (5 mm diameter, 0.256 cm^2^ electrode area, Pine Research Instrumentation), was polished with a 0.30 μm and a 0.05 μm Al_2_O_3_ polishing suspension (Bühler AG), sonicated in ultrapure water and subsequent cleaned by 1 M HClO_4_ and ultrapure water. Catalyst inks were carefully pipetted onto the electrode and dried at room temperature. Since the targeted platinum loading of the ink was always 0.08 mg_Pt_ cm^–2^, the pipetted ink volume was in the range of 14–20 μL. Electrochemical measurements were performed using an SP-150 BioLogic potentiostat and a rotator with a polyether ether ketone shaft (Pine Research Instrumentation) at room temperature. The counter electrode was a platinum mesh, and the reference electrode was a normal hydrogen electrode (HydroFlex, Gaskatel).

To determine the electrochemical surface area, *cyclic voltammetry* (*C–V*) was performed in a potential range of 0.05–0.8 V vs NHE with a scanning rate of 20 mV s^–1^ in a 0.1 M H_2_SO_4_ solution. Before the *C–V* measurements, we ran a cleaning cycle by cycling the potential from 0.0 to 1.3 V under an argon atmosphere at room temperature. The mean integral charge of the hydrogen adsorption areas was estimated, considering a charge of 210 μC cm_Pt_^–2^ for a full monolayer of hydrogen and a 0.77 monolayer at the onset of bulk hydrogen evolution.^28,29^ The indicated error is the standard deviation of 10 scans.

The oxygen diffusion coefficient was determined by using a 0.5 M H_2_SO_4_ solution as the electrolyte. The Pt loading of the catalyst layer was always 0.08 mg cm^2^. The solution was flushed with high-purity oxygen (O_2_) gas 20 min prior to the start of the experiment to ensure a saturated concentration of dissolved O_2_. During the measurement, a blanket of O_2_ gas was maintained over the surface of the solution to stabilize the concentration of dissolved oxygen. The potential was swept from 0.1 to 1.2 V vs NHE at 20 mV s^–1^ under a rotating rate of 100–1600 rpm. The rotating values were selected with a square root relationship for an intuitive representation of the Levich plot. The error stems from the linear regression of the Levich plot.

#### 2.5.3 Membrane Electrode Assembly (MEA)

The ink was deposited onto a Sigracet 22 BB gas diffusion layer via a spray coating (Prism 400 ultrasonic spray coater). The temperature of the heating was set to 80 °C when using D521 and to 90 °C when pPIM was used as an ionomer. The ink flow rate was set to 0.25 mL min^–1^ (with D521) and to 0.1 mL min^–1^ (with pPIM). Gas diffusion electrodes (GDE) with a loading of 0.4 mg_pt_ cm^–2^ for the cathode and a loading of 0.1 mg_pt_ cm^–2^ for the anode were fabricated. The exact loadings are given in Table S4. When using the pPIM as ionomer different I/C ratios were used: 0.2, 0.4, 0.63, and 0.8, corresponding to an ionomer-to-Pt/C ratio of: 0.08, 0.16, 0.25, and 0.32. An I/C ratio of 0.8 was used for the reference D521 catalyst ink. MEAs were prepared by the wet assembly method: we sandwiched a water-swollen commercial Nafion N211 membrane (25 μm) between the anode and cathode GDEs. In this stage of research, we are not able to achieve self-supported pPIM membranes to perform fuel cell tests due to their brittleness when cast. Therefore, a N211 membrane was used.

#### 2.5.4 Fuel Cell Testing

The MEAs were assembled in a balticFuelCells qCF FC25 liquid cooled single cell fixture with an active area of 25 cm^2^ (balticFuelCells GmbH). To ensure no gas leakage, two 50 μm PTFE gaskets were used on the anode side and one gasket on the cathode side. The active area compression was set to 3.3 bar_g_ via a pneumatic piston. The fuel cell hardware was mounted on a Greenlight Innovation G60 fuel cell test station equipped with a BioLogic VMP3 potentiostat. A leakage test was performed before every cell test. For conditioning, the cells were heated up to 80 °C, and the relative humidity was set to 96% at 2 bar_g_ pressure. Following, the H_2_/O_2_ supply was switched on, and the load was stepwise increased until 400 mA cm^–2^ was reached. The cell was conditioned under these parameters for 16 h. Polarization curves were obtained by applying a stepwise current sweep while recording the corresponding voltage response. Incremental steps of 0.02 A cm^–2^ were applied between 0 and 0.1 A cm^–2^ to accurately capture the activation region. Dwell times were limited to 30 s to minimize prolonged exposure to high potentials, which could lead to catalyst layer degradation. For current densities above 0.1 A cm^–2^, step increments of 0.1 A cm^–2^ were used, with dwell times of 60 s up to 0.4 A cm^–2^, and 120 s for all subsequent steps to ensure voltage stability.

Cyclic voltammetry was used to determine the electrochemically active surface area of the Pt catalyst with the CO-stripping technique, as described in previous literature.^30,31^ Diluted hydrogen (5% H_2_ in N_2_) was supplied to the counter electrode compartment (anode), and the working electrode compartment (cathode) was supplied with N_2_. Meanwhile, two cycles of *C–V* were scanned between 0.05 and 0.85 V to clean the cathode electrode surface. Afterward, dilute CO (1% CO in N_2_) was provided to the working electrode for 10 min at a constant potential of 0.125 V to form an adsorbed monolayer of CO on Pt. Any residual CO was removed by flushing the working electrode with N_2_ for 10 min. In the end, CO-stripping was carried out using two cycles of *C–V* between 0.05 and 0.85 V with a sweep rate of 50 mV s^–1^ (see Figure S10). The two cycles were repeated three times, and the standard deviation was calculated.

The ionic resistance of the ionomer within the catalyst layer was analyzed using electrochemical impedance spectroscopy (EIS) under H_2_/N_2_^32^ at a potential of 0.5 V to avoid contributions from Faradaic currents (see Figure S11).^33^ A finite transmission line model was used to determine the ionic resistance of the cathode catalyst layer from the impedance response.^34^ The Warburg-like impedance response, characterized by a 45° slope in the high-frequency region, is derived from proton migration through the ionomer within the catalyst layer. Under N_2_ conditions at the cathode, the limiting capacitance response manifests as a vertical line, representing the theoretically infinite kinetic resistance. The ionic resistance of the cathode catalyst layer (*R*_CCL_) was determined by projecting the length of the Warburg-like region onto the real impedance axis (Z′): .

For the analyses of the oxygen diffusion coefficient at the cathode catalyst layer (CCL), EIS measurements at a low current density of 40 mA cm^–2^ were performed at two different oxygen concentrations in the cell, as reported by Kulikovsky.^35^ A relation between *D*_O2_ and the difference between the cathode catalyst layer area specific resistances, δ*R*_CCL_, corresponding to the two oxygen concentrations, 100 and 50% O_2_ in N_2_, is used.

#### 2.5.5 Scanning Electron Microscope (SEM)

Embedded cross-sectional images of tested MEAs have been taken to determine the catalyst layer thickness for the analysis of the oxygen diffusion coefficient. The MEAs were sandwiched between two PTFE sheets, embedded in epoxy resin (Epo Thin, Buehler), and cured overnight under vacuum at room temperature. Subsequently, the samples were ground successively from 220 to 4000 grit sizes with silicon carbide grinding paper (Struers GmbH) and polished with a MD-Mol polishing plate using a 3 μm diamond polishing paste (ATM GmbH). The polishing machine LaboForce-100 (Struers GmbH) was used for grinding and polishing. To achieve better electric conductivity, a thin gold film was sputtered onto all samples using a gold sputter coater (108 Manual Sputter Coater, Cressington). For the SEM measurements, a Tescan Vega 3 SEM with an accelerating voltage of 20 kV and a secondary electron detector was used.

## 3 Results and Discussion

### 3.1 Synthesis and Polymer Characterization

**Figure 1:**
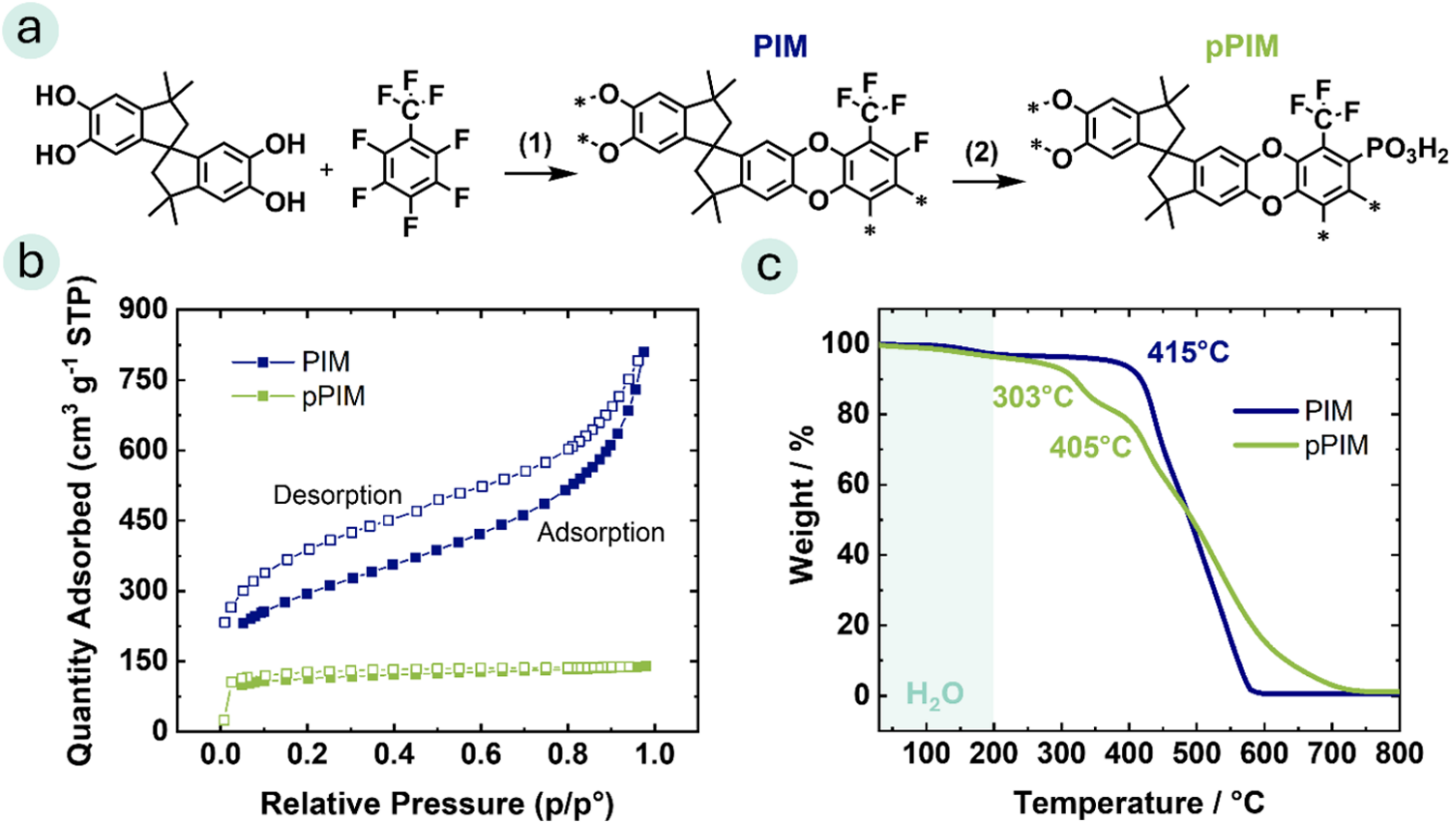
(a) Scheme of the synthetic pathway to phosphonated pPIM: (1) Polycondensation and (2) S_N_Ar Michaelis Arbuzov reaction. (b) N_2_ adsorption/desorption isotherms at 77 K, and (c) thermogravimetric analysis in a synthetic air atmosphere with a heating rate of 10 °C min^–1^ of PIM and pPIM.

The polymer with intrinsic microporosity was synthesized using S_N_Ar polycondensation following reaction procedures similar to those reported in previous literature (Figure 1a).^36^ A polymerization temperature of 160 °C and a monomer concentration of 0.5 M (monomer:solvent = 1 mmol:2 mL) in DMAc/toluene were applied. A DMAc/toluene (2:1 v/v) solution was used to dissolve the monomers homogeneously and to provide solubility of the formed polymer. The ideal reaction time was found to be 62 min. Allowing the reaction to stir for a longer period resulted in the formation of excessively cross-linked polymer structures, which can be seen by the increase in polydispersity (see Table S1). Compared with the literature,^36,37^ the fluorinated monomer reported in this study has five reactive sites, making the polycondensation difficult to control. Consequently, polymers with molecular weights of Mn = 15.4 kg mol^–1^, Mw = 102.1 kg mol^–1^, and PDI = 6.6 (see Figure S2) were obtained. Konno et al.^38^ reported a cross-linking approach in which fluorinated monomers react nonlinearly, forming a complex polymer network rather than a linear polymer. Another side reaction that might happen is the cyclization of the polymer, which is described in the study of Kricheldorf.^39^ Notably, prolonging the reaction time to 24 h at room temperature did not yield polymers with higher molecular weights or lower polydispersity. We assume that the initial nucleophilic attack occurs at the para-fluorine atom (C–4)^40−42^ followed by substitution at the meta fluorine atom (C–3) due to the close proximity of the second nucleophilic oxygen of the spiro molecule. In the third step, nucleophilic attack is expected at the C–6 position, as the trifluoromethyl group and ether linkages will direct toward this site, while the C–2 position is sterically hindered by the spiro molecule. Finally, substitution occurs at the fluorine atom in the C–5 position (see Figure S3). Proton conductive groups were introduced via the S_N_Ar Michaelis Arbuzov reaction.^40,43,44^ In this work, a phosphonation was used, as sulfonation may lead to cross-links between sulfonic acid groups, which can reduce proton conductivity.^45^ Notably, the neighboring trifluoromethyl group, with its electron-withdrawing effect, enhances the acidity of the phosphonic acid group. According to ^19^F-NMR and ^31^P NMR the phosphonation degree is 59%. The signal-splitting of the trifluoromethyl group in ^19^F-NMR confirms the substitution of the neighboring fluorine atom (Figure S4). In addition, the substitution degree was calculated back from the ion exchange capacity, which was determined via salt splitting titration at room temperature. The IEC_direct_ was found to be 0.75 ± 0.03 mequiv g^–1^ and the IEC_total_ 1.46 ± 0.05 mequiv g^–1^, resulting in an overall phosphonation degree of 38%. The difference in the phosphonation degree between the two determination methods is referred to as “dead ends” in the ion channels mitigating the IEC value. However, the IEC value is comparable with PIMs having similar polymer structures, which exhibit an IEC of 1.68 mequiv g^–1^.^18^ Partial phosphonation was also confirmed through Fourier Transform Infrared (FT-IR) spectroscopy (Figure S5). The FT-IR spectrum of the phosphonated PIM exhibits relatively broad and weak bands, which can be assigned to the stretching vibration of hydrogen bonds in (P)O–H at 2500–3600 cm^–1^. Stretching vibrations of PO_3_ give rise to the new absorption band at 1436 cm^–1^. The weak new absorption band at 1046 cm^–1^ was assigned to stretching vibration of P = O.^43^

Nitrogen adsorption and desorption were analyzed to investigate the pore structures of the PIM materials. In Figure 1b, the pristine, unphosphonated PIM exhibits desorption hysteresis with a decreased BET surface area measured during the adsorption phase, while the pPIM exhibits a nearly ideal Langmuir isotherm during the adsorption and desorption phases. This suggests that the pristine PIM encounters a kinetic barrier to adsorption, whereas the pPIM demonstrates improved pore connectivity, enhancing the efficiency of gas transport.^18^ The pristine PIM shows a BET surface area of 1260 ± 290 m^2^ g^–1^, while the phosphonated pPIM has a lower value of 420 ± 4 m^2^ g^–1^. This may be explained by the introduction of phosphonic acid groups, which enable several interactions such as hydrogen bonding and ion-dipole interactions between polymer chains. The stronger interaction between polymer chains in the pPIM leads to reduced interchain distances and polymer domain spacing, thereby resulting in decreased BET surface areas.^18^ The obtained BET surface areas are in good agreement with values reported in previous literature.^18,19,37^ Thermogravimetric analyses revealed the high thermal stability of the polymer materials, depicted in Figure 1c. The first weight loss observed up to a temperature of 200 °C for the pristine PIM and the phosphonated pPIM is attributed to water residues in the polymer matrix. The second weight loss step of the phosphonated pPIM at an onset temperature of 303 °C is due to dephosphonation, while the third step is related to the degradation of the polymer backbone (*T*_onset_ = 405 °C). The pristine PIM showed an onset decomposition temperature of 415 °C. In differential scanning calorimetry measurements, the glass transition temperature of PIM and pPIM was not discernible (see Figure S6). This observation aligns well with previously reported literature.^18^

### 3.2 Characterization as Binder Material

Rotating disk electrode measurements were used to investigate the impact of the solvents used in the ink formulation on the ECSA. DMF or DMAc were utilized to dissolve the pPIM, while various solvents were employed for the overall ink solution, as summarized in Table S2. The highest ECSA of 39.6 ± 3.4 m^2^ g^–1^ was achieved using DMAc as the polymer solvent and a mixture of 15 wt % H_2_O in DMAc as the ink solvent. This is likely due to the excellent miscibility of the components, which prevents polymer precipitation. Consequently, these solvents were selected for further ink studies with pPIM as the ionomer. For the reference ink, a Nafion D521 dispersion with a solvent mixture of 40 wt % in 1-propanol was found to be optimum, reaching an ECSA of 42.1 ± 1.4 m^2^ g^–1^.

**Figure 2:**
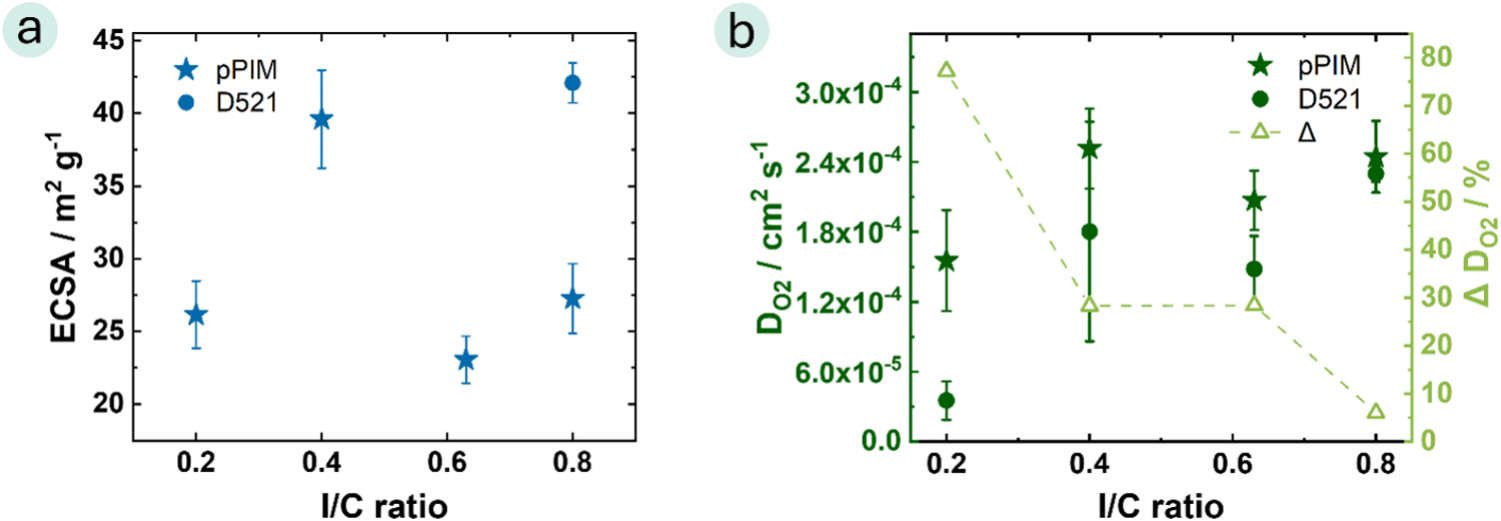
(a) ECSA values (Atmosphere: Ar, *C–V*: 0.05–0.8 V, no rotation, scanning rate: 20 mV s^–1^, electrolyte: 0.1 M H_2_SO_4_ with *C–V* cleaning 0.0–1.3 V); (b) Oxygen diffusion coefficient of catalyst layers with pPIM or D521 at different I/C ratios using RDE measurements (Atmosphere: O_2_, *C–V*: 0.1–1.2 V scanning rate: 20 mV s^–1^, electrolyte: 0.5 M H_2_SO_4_) and delta D_O2_, representing the percentage difference between the pPIM and D521 catalyst layers.

To achieve optimal fuel cell performance, it is crucial to balance the I/C ratio, which enhances the proton conductivity, improves catalyst utilization, and ensures efficient access to reactant sites. Higher I/C ratios can enhance the proton conductivity and improve the structural stability by ensuring adequate coverage of platinum catalyst particles. However, excessively high I/C ratios can negatively affect the performance of the catalyst. Excess ionomer can fill the pores in the catalyst layer, or it can lead to increased water uptake, reducing gas diffusion pathways and hindering the accessibility of active sites for reactants.^46^ Different I/C ratios (0.2, 0.4, 0.63, and 0.8) of the pPIM ionomer ink were therefore investigated. RDE measurements revealed a maximum ECSA of 39.6 ± 3.4 m^2^ g^–1^ when using an I/C ratio of 0.4, approaching the ECSA of 42.1 ± 1.4 m^2^ g^–1^ measured for the D521-based catalyst layer with an I/C ratio of 0.8 (Figure 2a).

Because of their porous microstructure, PIMs are expected to exhibit enhanced oxygen diffusivity. This property was examined through RDE measurements employing the Koutecky–Levich (KL) method,^47^ according to which the disc current (*i*_d_) on a rotating disc electrode can be calculated using the equation:
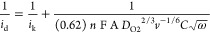
where *i*_k_ is the kinetic current of the ORR, *n* is the number of electrons involved in the reaction (4 e^–^),^48^ F is the Faraday’s constant (96485 C mol^–1^), A is the area of the disc electrode (0.256 cm^2^), *D*_O2_ is the diffusion coefficient of oxygen (cm^2^ s^–1^), *v* is the kinematic viscosity (0.01 cm^2^ s^–1^), *C* the solubility of oxygen (1.10 × 10^–6^ mol cm^–3^), and ω is the electrode angular rotation rate (rad s^–1^).^49^

Herein, the ORR was investigated by measuring the current at the Pt disc working electrode as the voltage is swept from 0.1 to 1.2 V vs NHE at a scan rate of 20 mV s^–1^ with different electrode rotation rates, 100, 400, 900, and 1600 rpm, respectively (Figure S7). At the mass transfer limited region (herein approximately at *E* = 0.4 V), the rate of the ORR reaction is limited by the availability of oxygen at the surface, and the Levich plot follows a linear relationship:
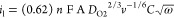


By plotting *i*_l_ (limited current) against ω^1/2^ and performing linear regression, we can use the obtained slope (m) to determine the diffusion coefficient of oxygen:
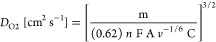


We assume a four-electron transfer occurs, where O_2_ is directly reduced to H_2_O.^48^ The concentration of O_2_ in the electrolyte is considered equivalent to its solubility, indicating that the solution is fully saturated.

Overall, it can be concluded that the pPIM-based catalyst layers exhibit a higher oxygen diffusion compared with the D521-based catalyst layers for all I/C ratios. The highest oxygen diffusion coefficient was measured for the pPIM ink with an I/C ratio of 0.4 (*D*_O2 pPIM 0_._4_ = 2.5 × 10^–4^ ± 3.4 × 10^–5^ cm^2^ s^–1^). The higher oxygen diffusion coefficient underlines the formation of a porous polymer microstructure. Interestingly, when comparing the percentage difference in oxygen diffusion coefficients between pPIM and D521 (Figure 2b), we observe that the difference is largest for I/C ratios of 0.2, and that difference gradually diminishes becoming nearly identical at 0.8. This difference may be attributed to the lower gravimetric density of hydrocarbon ionomers (in the range of 1.3 g cm^–3^)^50^ compared to fluorinated ionomers (1.9 g cm^–3^ for Nafion).^51^ In hydrocarbon-based ionomers, a lower gravimetric ionomer content is sufficient to achieve adequate catalyst coverage, striking a balance between ionic resistance, complete catalyst coverage, and effective oxygen diffusion. It appears that as the I/C ratio is increased, the ionomer layer becomes increasingly thick that the increased pPIM porosity no longer has a significant influence on the oxygen diffusion coefficient. Previous studies have indeed demonstrated that improved fuel cell performance is achieved at lower gravimetric ionomer contents when using hydrocarbon ionomers, which is in contrast to the behavior observed with Nafion-based ionomers.^52,53^

**Figure 3:**
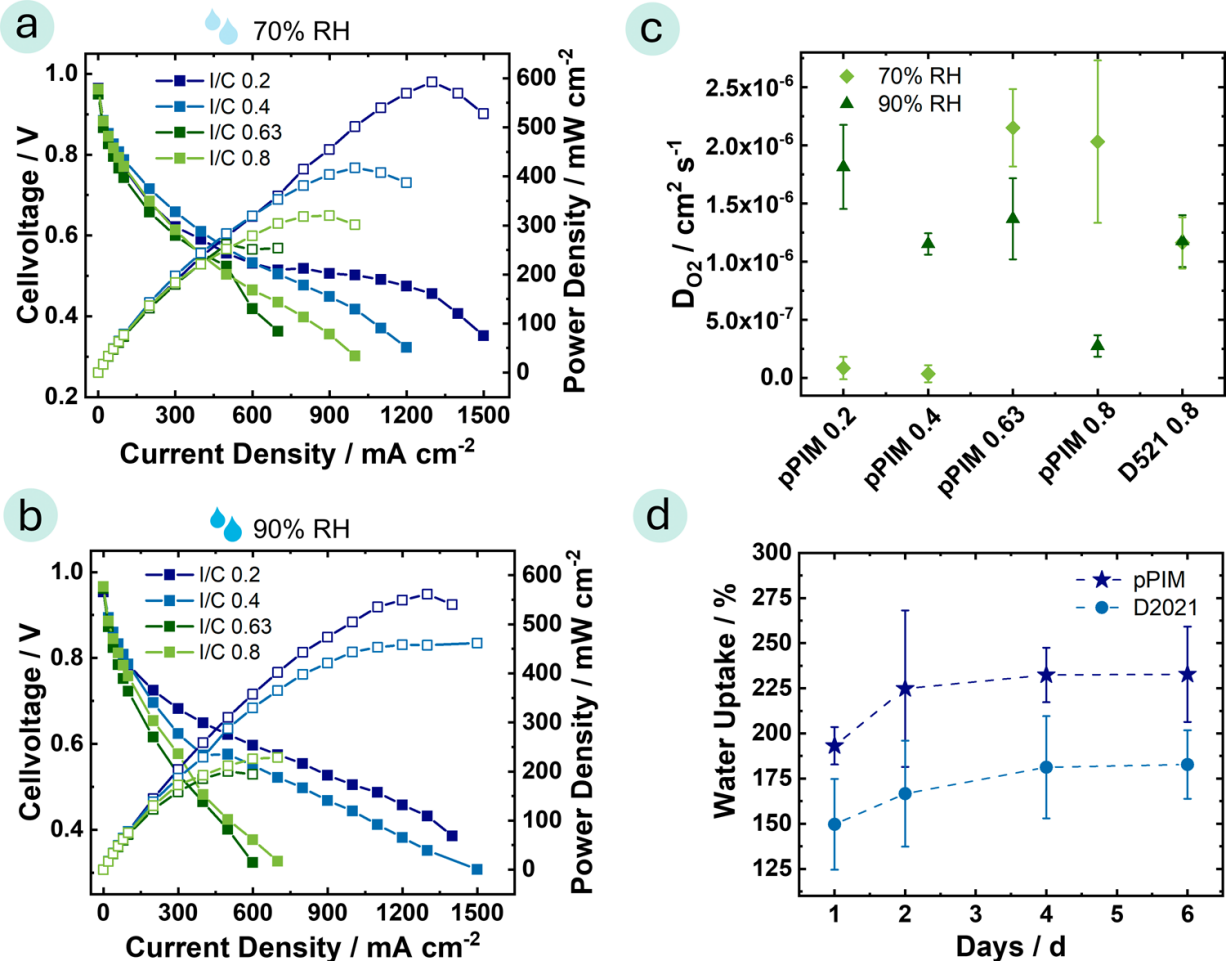
Polarization (filled squares—left *y*-axis) and power density (open squares—right *y*-axis) curves of MEAs comprising of a N211 membrane and a catalyst layer with pPIM as ionomer with different I/C ratios at 80 °C, 2 bar-a, under H_2_/O_2_ condition with a cathode loading of 0.4 mg_pt_ cm^–2^ and an anode loading of 0.1 mg_pt_ cm^–2^ (exact loadings in Table S4) at (a) relative humidity of 70% and (b) of 90%, (c) corresponding oxygen diffusion coefficient values (the error bars are calculated with error propagation taking into account the standard deviation of the catalyst layer thickness determination via SEM and the standard deviation of measuring three pEIS). (d) Water uptake of pPIM membrane pieces and the D2021 membrane at 85 °C (the error bars represent the standard deviation of three samples).

The single cell PEMFC performance of the pPIM binders was investigated at relative humidities of 70% and 90%, and an operation temperature of 80 °C (see Figure 3a,b). To investigate the influence of the ionomer content on the overall fuel cell performance, I/C ratios of 0.2, 0.4, 0.63, and 0.8 were chosen. Overall, the best performance was observed at an I/C ratio of 0.2, achieving a peak power density of 593 mW cm^–2^ at a moderate relative humidity of 70% RH. When the relative humidity was increased to 90% RH, the peak power density was reduced slightly to 561 mW cm^–2^. Based on these results, we can assume that an I/C ratio of 0.2 provides sufficient ionomer coverage of the catalyst to ensure adequate reactant supply to the catalyst surface while avoiding excessive ionomer swelling that may lead to the obstruction of active sites. The higher peak power density at 70% RH underlines the influence of water uptake and swelling of the ionomer material on the fuel cell performance. Conversely, at higher I/C ratios, the swelling of the ionomer increases, thus blocking more active sites and resulting in a decreased fuel cell performance.

The oxygen diffusion coefficient at the cathode catalyst layer in the fuel cell setup was determined using the method described by Kulikovsky.^35^ EIS measurements at a low current density of 40 mA cm^–2^ were performed at two different oxygen concentrations in the cell (100 and 50% O_2_ in N_2_). An exemplary pEIS is presented in Figure S14. The oxygen diffusion coefficient can be calculated using the following equation:
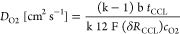
where k is the ratio of oxygen concentrations of the two experiments, b is the Tafel slope (V), *t*_CCL_ is the cathode catalyst layer thickness (cm) (determined by SEM cross-section measurements of the MEA), F is the Faraday constant (96485 C mol^–1^), δ*R*_CCL_ is the difference between the CCL area specific resistances of the two experiments (Ω cm^2^), and *c*_O2_ is the oxygen concentration at the CCL/GDL interface (mol cm^–3^). Figure 3 shows the *D*_O2_ plotted as a function of I/C, where we observe that for I/C ratios of 0.2 and 0.4, the oxygen diffusion coefficient increases with increasing relative humidity. The *D*_O2_ with an I/C ratio of 0.2 is 8.5 × 10^–8^ ± 9.6 × 10^–8^ cm^2^ s^–1^ at 70% RH and is 1.8 × 10^–6^ ± 3.6 × 10^–7^ cm^2^ s^–1^ at 90% RH. We assume that higher relative humidity leads to increased water content and swelling of the ionomer, leading to an expansion of its hydrophilic ionic domains. These findings also agree with studies indicating that oxygen diffuses faster in the hydrophilic domains of the ionomer.^14,15^ In contrast, at I/C ratios of 0.63 and 0.8, the oxygen diffusion coefficient decreases as relative humidity is increased (Figure 3c). The oxygen diffusion coefficients at 70% RH are D_O2 pPIM 0.63_ = 2.2 x10^–6^ ± 3.3 × 10^–7^ cm^2^ s^–1^, D_O2 pPIM 0.8_ = 2.0 × 10^–6^ ± 6.9 × 10^–7^ cm^2^ s^–1^, whereas at 90% RH D_O2 pPIM 0.63_ = 1.4 × 10^–6^ ± 3.5 × 10^–7^ cm^2^ s^–1^, and D_O2 pPIM 0.8_ = 2.7 × 10^–7^ ± 9.2 × 10^–8^ cm^2^ s^–1^. To summarize the overall trend, we observe that high relative humidity conditions favor increased oxygen diffusion when a low I/C ratio is used but that oxygen diffusion is increased under lower relative humidity conditions when a high I/C ratio is used. This suggests that while oxygen diffusion is faster in hydrophilic domains, there is a limit at higher I/C ratios. At higher I/C ratios, it appears that gas transport is primarily dominated by the availability of free volume within the catalyst layer.^54^

A comparison of the overall fuel cell performance with the oxygen diffusion coefficient reveals a clear correlation that higher oxygen diffusion coefficients correspond to better fuel cell performance. The exception to this trend is observed at an I/C ratio of 0.2. These results emphasize the importance of tailoring the I/C ratio and ionomer properties, such as water uptake and oxygen diffusion, to provide optimal fuel cell performance.

**Figure 4:**
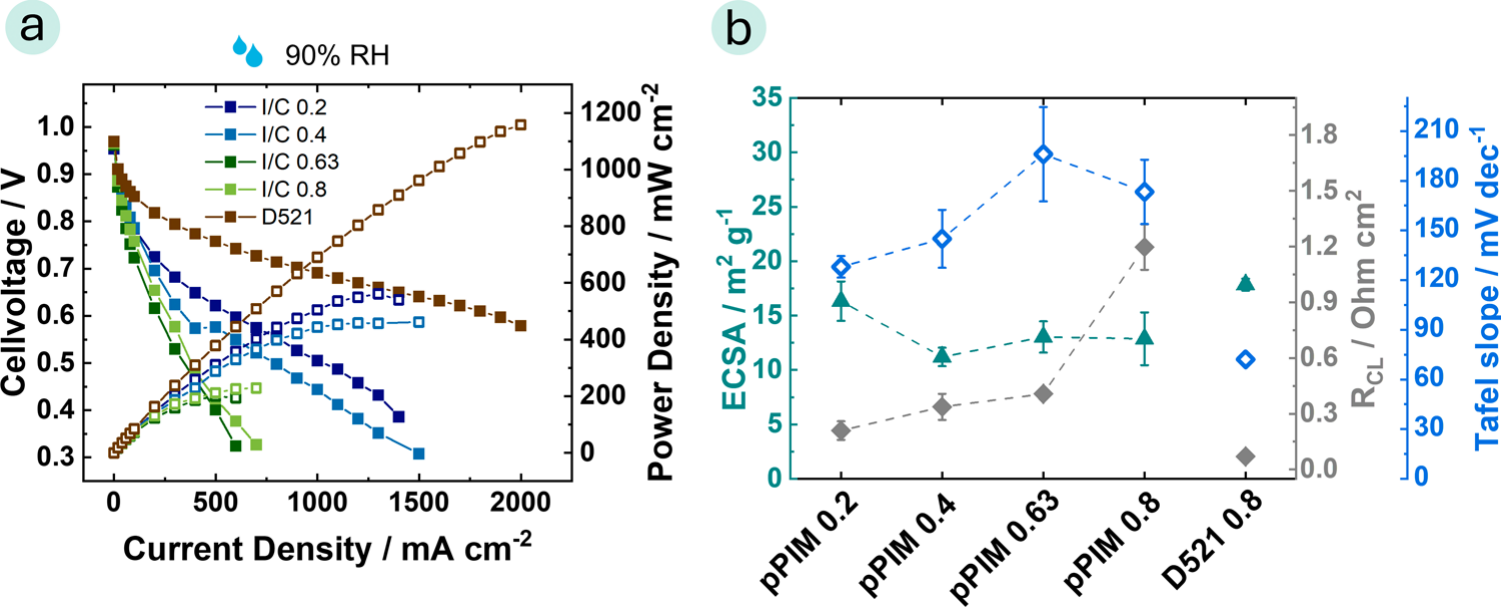
(a) Polarization (filled squares—left *y*-axis) and power density (open squares—right *y*-axis) curves of MEAs comprising of a N211 membrane and a catalyst layer with pPIM as ionomer with different I/C ratios and using D521 as a reference ionomer at 80 °C, 2 bar-a, under H_2_/O_2_ condition with a cathode loading of 0.4 mg cm^–2^ and an anode loading of 0.1 mg cm^–2^ (exact loadings in Table S4) at relative humidity of 90%, (b) corresponding ESCA (triangle—left *y*-axis), protonic sheet resistance (filled squares—right *y*-axis) and Tafel slope values (open squares—right *y*-axis).

To compare the performance of pPIM with that of state-of-the-art material, a reference measurement using D521 as a binder material in the catalyst layer was conducted. The MEA with D521 shows the best performance, which is also visible from the ECSA, ionic resistance of the cathode catalyst layer (*R*_CL_), and Tafel slope values plotted in Figure 4b. Using D521 as the binder material in the catalyst leads to an ECSA of 17.8 ± 0.5 m^2^ g^–1^, *R*_CL_ of 70 ± 5 mΩ cm^2^, and a Tafel slope of 72 mV dec^–1^. For the pPIM, an I/C ratio of 0.2 achieved the highest fuel cell performance with an ECSA of 16.3 ± 1.8 m^2^ g^–1^, *R*_CL_ of 210 ± 50 mΩ cm^2^, and a Tafel slope of 128 mV dec^–1^. One possible explanation for the lower ECSA may be its higher water uptake compared to that of the Nafion-based ionomer (see Figure 3d). After 1 day at 85 °C, the pPIM material shows a water uptake of 193 ± 10%, whereas the Nafion-based material exhibits a water uptake of 149 ± 25%. The greater swelling of the ionomer in the catalyst layer can result in the blocking of active sites. Observed differences between ionomers are typically closely related to activation, ohmic, and transport losses. Therefore, to better elucidate these losses for the catalyst layers that contained PFSA or pPIM binder, we plotted the ohmic polarization loss (*iR*)-free cell potential (Figure S9) and found that the remaining cell potential is dominated by differences in mass transport resistances. This is expected as ohmic losses are usually dictated by the proton conductivity of the PEM, and in this study, we used the same N211 PEM in all fuel cell tests. Differences in mass transport resistances may be explained by the superior water management properties of the D521 binder. Greater swelling observed for the pPIM binder may result in localized flooding, adversely affecting the performance. Additionally, the better performance of the D521 binder may be attributed to the better interfacial compatibility with the N211 membrane. This is witnessed by the improved contact resistance between the membrane and the catalyst layer. Cross-sectional SEM measurements of the MEAs reveal better adhesion between the N211 membrane and the catalyst layer with the D521 binder (see Figures S12 and S13). During the embedding process, the D521 catalyst layer remained firmly attached to the membrane, whereas the catalyst layer with the pPIM binder showed detachment. This may indicate weaker adhesion to the membrane, leading to increased ohmic resistance of the MEA and diminished fuel cell performance.^55^

**Figure 5:**
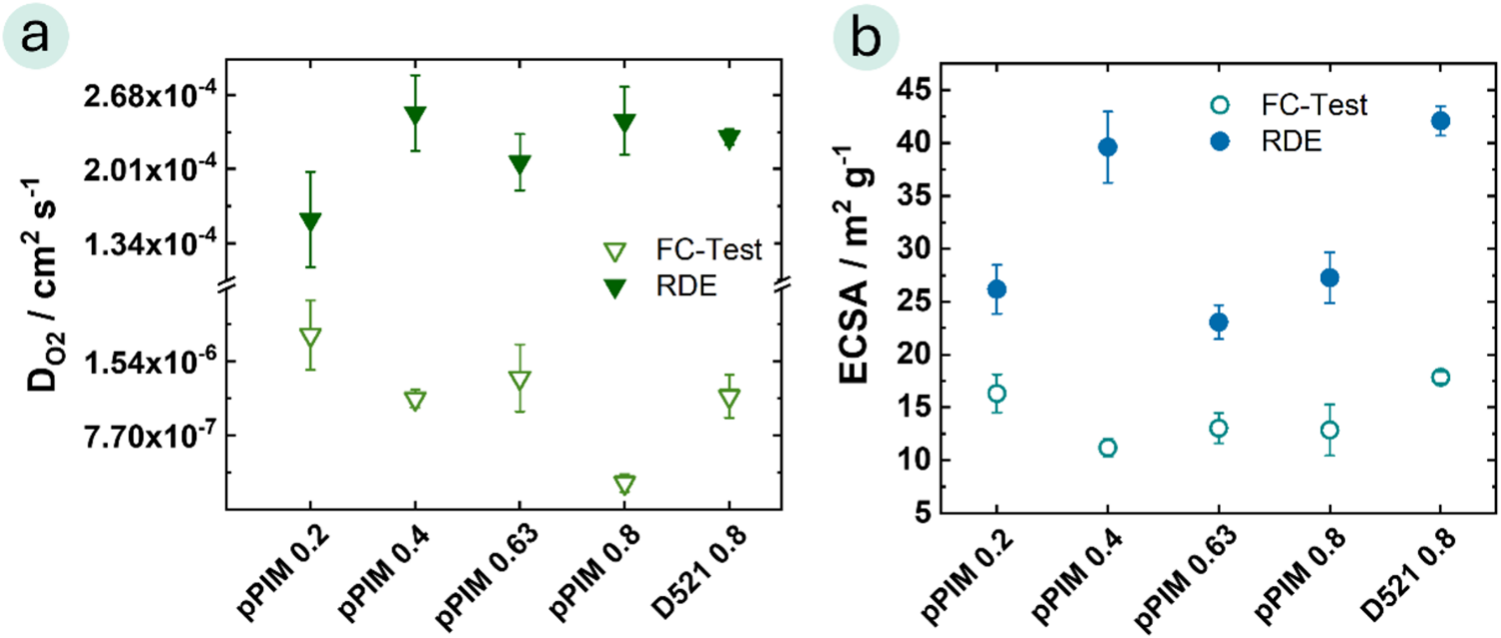
Comparison of RDE and FC measurements in regard to (a) oxygen diffusion coefficient and (b) ECSA values. (FC test at RH = 90%, 80 °C, 2 bar-a, under H_2_/O_2_ condition with a cathode loading of 0.4 mg_Pt_ cm^–2^ and an anode loading of 0.1 mg_Pt_ cm^–2^).

Finally, the FC and RDE measurements of ESCA and the oxygen diffusion coefficient were compared in Figure 5. Overall, a lower oxygen diffusion coefficient was observed in the FC measurements. Furthermore, in the RDE measurements, the highest oxygen diffusion coefficient was obtained with an I/C ratio of 0.4, whereas the FC test showed the highest oxygen diffusion coefficient at an I/C ratio of 0.2. The same trend applies to the ECSA comparison. Nevertheless, in the FC measurement, the pPIM catalyst with an I/C ratio of 0.2 shows a higher oxygen diffusion coefficient (D_O2 pPIM 0.2_ = 1.8 × 10^–6^ ± 3.6 × 10^–7^ cm^2^ s^–1^) compared to the reference catalyst layer with D521 (*D*_O2 D521 0.8_ = 1.2 × 10^–6^ ± 2.2 × 10^–7^ cm^2^ s^–1^) and its ECSA is in the same range (ECSA _pPIM 0.2_ = 16.3 ± 1.8 m^2^ g^–1^, ECSA _D521 0.8_ = 17.8 ± 0.5 m^2^ g^–1^). It is worth noting that ECSA values determined from both in situ operation and RDE measurements can be equally valuable. However, it is essential to consider the systematic errors inherent to each method.^28,31,56^ Previous literature indicates that even when using commercial samples, the determination of ECSA through CO-stripping exhibits a notable error.^30^ This may explain the deviation between the ESCA values derived from RDE and fuel cell measurements.

## 4 Conclusions

To conclude, we presented a facile, two-step synthesis of a novel polymer of intrinsic microporosity featuring proton conductive phosphonic acid groups. The polymer shows a high thermal stability and a high BET surface area. Furthermore, we used the novel pPIM as a catalyst layer ionomer in fuel cell studies and systematically varied the I/C ratio in the catalyst inks in order to gain valuable insights into its effect on the oxygen diffusion coefficient. At an optimal I/C ratio of 0.2, the pPIM ionomer exhibits improved oxygen diffusion and enhanced ECSA. At this ionomer-to-carbon ratio, the catalyst coverage remains sufficient, and ionomer swelling does not hinder reactant access to active sites. To address the brittleness of the pPIM polymer, future research will focus on modifying the fluorinated monomer. One approach is to use hexafluorobenzene, functionalized with phosphonated or sulfonated groups at positions 1 and 4-positions. By ensuring that only four fluorine atoms participate in the polymerization, the reaction can be more precisely controlled, leading to higher molecular weights. Furthermore, prefunctionalizing the monomer may ensure sufficiently high IEC, as two acid groups per unit are present in the polymer backbone. Nevertheless, this study highlights the significance of optimizing the I/C ratio in the catalyst layer as well as ionomer properties, such as microstructure and water uptake, to improve fuel cell performance. Overall, the microporous structure of pPIMs enhances oxygen diffusion, making them promising candidates to be used as ionomers in the catalyst layers of PEMFCs.

## Supporting Information Available

GPC data of PIM, scheme of PIM synthesis, ^19^F-NMR and ^1^H NMR of PIM, molar mass profile of PIM, directing effects of PIM synthesis, scheme of pPIM synthesis, ^19^F-NMR. ^1^H NMR and ^31^P NMR of pPIM, FT-IR spectra of PIM and pPIM, DSC curve of PIM and pPIM, table of RDE solvent study, RDE measurements of pure polymer solutions, current potential curves, H_2_/N_2_ EIS spectra, SEM cross sections of tested MEAs with EDX analysis, polarization curves at 70% RH and 90% RH with HFR values, *iR*-free polarization curves, CO-stripping voltammograms, anode and cathode Pt loadings, and pEIS (PDF)

## Abbreviations

PEMFC: proton exchange membrane fuel cell
PEM: proton exchange membrane
PIM: polymer of intrinsic microporosity
pPIM: phosphonated polymer of intrinsic microporosity
MEA: membrane electrode assembly
CL: catalyst layer
CCL: cathode catalyst layer
GDL: gas diffusion layer
R_CCL_: area specific resistance of the cathode catalyst layer
R_CL_: ionic resistance of the cathode catalyst layer
GDE: gas diffusion electrode
ORR: oxygen reduction reaction
IEC: ion exchange capacity
NMR: nuclear magnetic resonance
GPC: gas permeation chromatography
BET: Brunauer-Emmett-Teller
TGA: thermogravimetric analysis
FT-IR: Fourier transform infrared
RDE: rotating disc electrode
ECSA: electrochemically active surface area
WU: water uptake
SEM: scanning electron microscopy
*C–V*: cyclic voltammetry
EIS: electrochemical impedance spectroscopy
D_O2_: oxygen diffusion coefficient
Pt: platinum
PTFE: poly(tetrafluoroethylene)
S_N_Ar: aromatic nucleophilic substitution reaction
I/C: ionomer-to-carbon
RH: relative humidity
