# Supplementary material for: Fluorine-Lean Phosphonated Polymers of Intrinsic Microporosity with High Oxygen Permeability as a PEMFC Catalyst Layer Ionomer
Source: ACS Appl Energy Mater. 2025 Mar 28;8(7):4629–39. doi: 10.1021/acsaem.5c00265 (PMC12001289; doi:10.1021/acsaem.5c00265)
Supplement: Supplementary file 1 — ae5c00265_si_001.pdf [file ae5c00265_si_001.pdf]

Supporting Information

Fluorine-Lean Phosphonated Polymers of Intrinsic Microporosity with High Oxygen Permeability as PEMFC Catalyst Layer Ionomer

Theresa Stigler [a,b], *Tamas Nemeth* [c], *Patrick Fortin* [c], *Simon Thiele* [a,b], *Jochen Kerres* [a,d]*

a. Forschungszentrum Jülich GmbH, Helmholtz Institute Erlangen-Nürnberg for Renewable Energy (IET-2), Cauerstr. 1, 91058 Erlangen, Germany.

b. Department of Chemical and Biological Engineering, Friedrich-Alexander-Universität Erlangen-Nürnberg, Immerwahrstr. 2a, 91058 Erlangen, Germany.

c. Department of Sustainable Energy Technology, SINTEF Industry, 7034 Trondheim, Norway.

d. Chemical Resource Beneficiation Faculty of Natural Sciences, North-West University, Potchefstroom 2520, South Africa.

*Corresponding author, E-Mail: j.kerres@fz-juelich.de, th.stigler@fz-juelich.de.

Table S1. GPC measurements of the PIM polycondensation at different reaction times.

Time / min	Mw / kg mol^{-1}	Mn / kg mol^{-1}	PDI
**60**	44.5	15.3	2.9
**65**	123.4	21.7	5.7
**69**	190.9	24.6	7.7
**73**	266.0	20.3	14.6

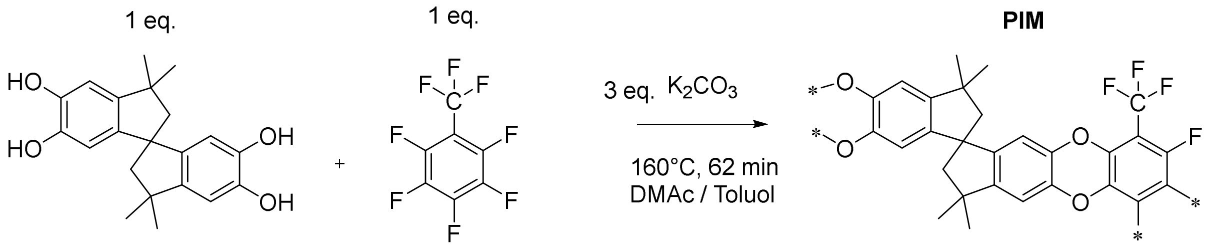

Scheme S1. Polycondensation of PIM.

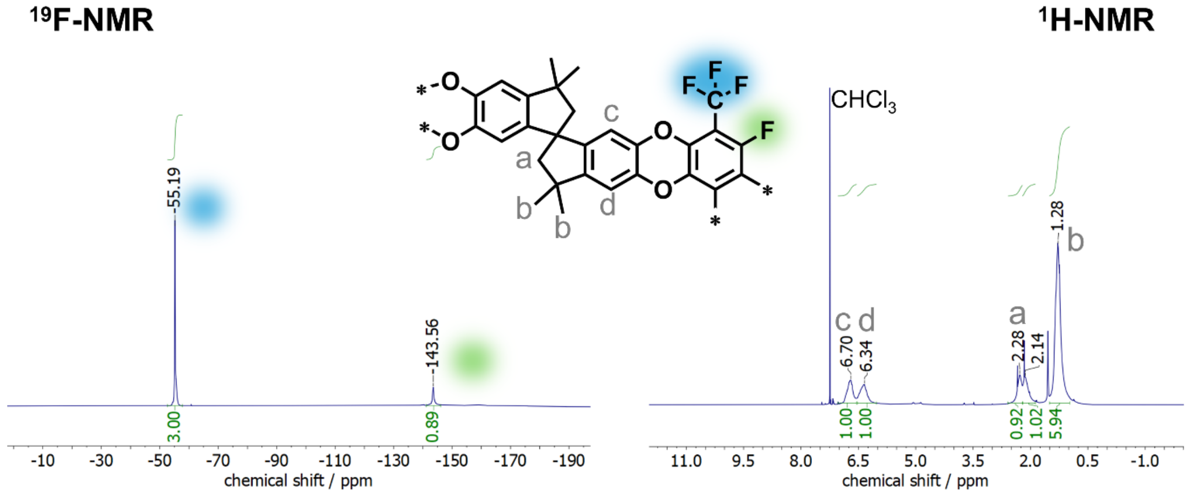

Figure S1. ^{19}F-NMR and ^{1}H-NMR of PIM with the respective alignment of the resonances (solvent: $CDCl_3$).

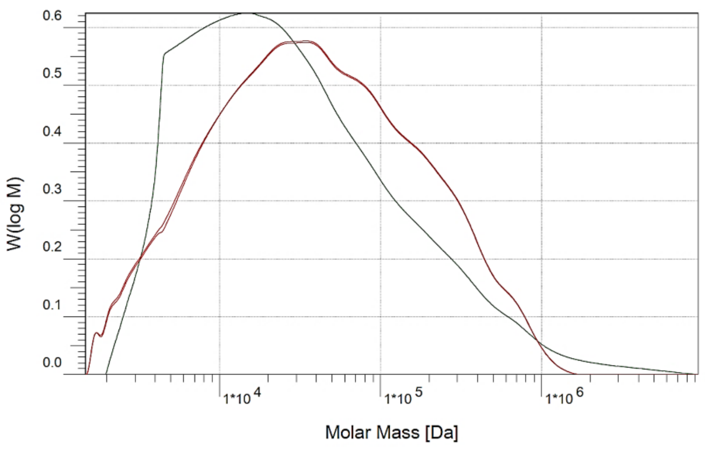

Figure S2. Molar mass profile of PIM after a reaction time of 62 min for the upscaled polymerization (eluent: THF, T:35°C, standard: polystyrene): Mn = 15.4 kg mol^{-1}, Mw = 102.1 kg mol^{-1}, PDI 6.6.

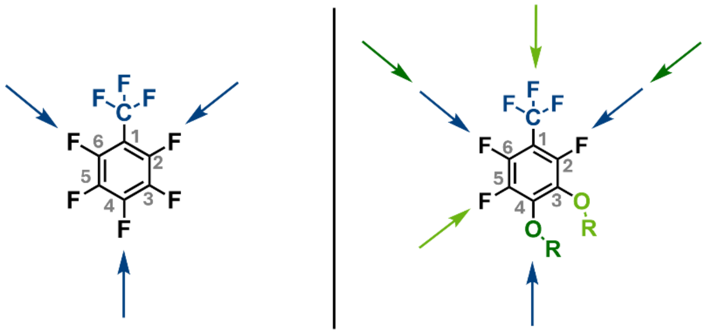

Figure S3. Directing effects of functional groups with regards to the nucleophilic aromatic substitution for the polycondensation reaction.

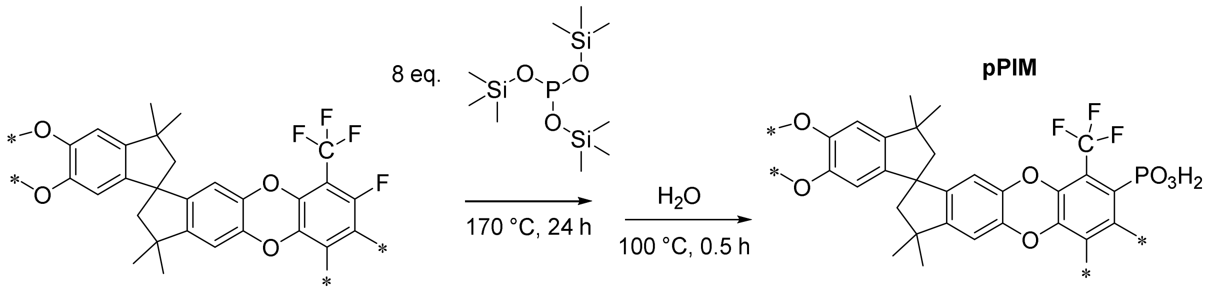

Scheme S2. S_NAr Michaelis Arbuzov reaction of pPIM.

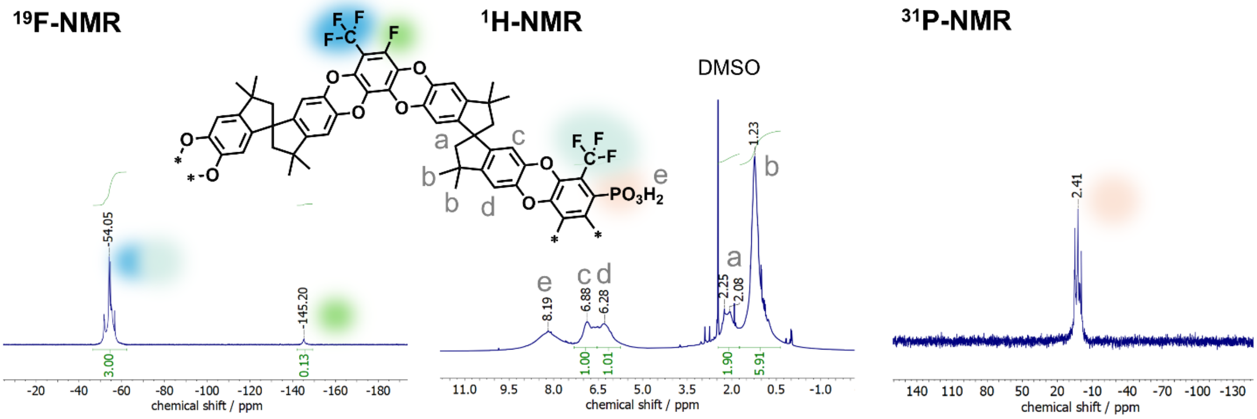

Figure S4. ^{19}F-NMR,^{1}H-NMR and ^{31}P-NMR of pPIM with the respective alignment of the resonances (solvent: d_6-DMSO).

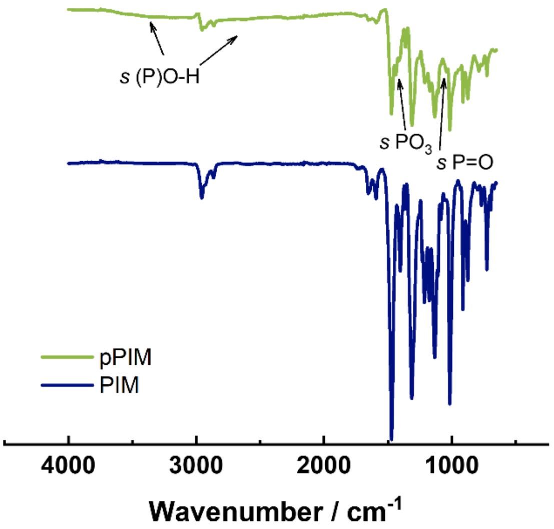

Figure S5. FT-IR spectra of PIM and pPIM.

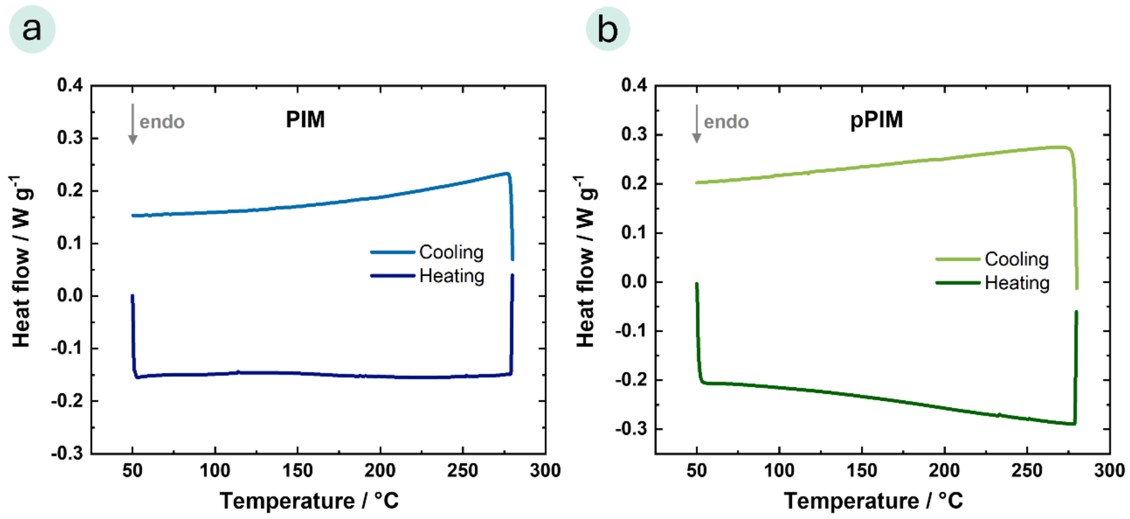

Figure S6. DSC curve of (a) pristine PIM and (b) phosphonated pPIM (heating rate = 10 K min^{-1}, nitrogen atmosphere, flow rate = 50 mL min^{-1}).

Table S2. RDE study of different solvents used for the catalyst ink and the respective ECSA values.

Solvent Ionomer	Polymer	Solvent Ink	I/C ratio	ECSA $m^2\,g^{-1}$
DMF	pPIM	15 wt% H_2O in EtOH	0.4	28.0 ± 2.4
DMF	pPIM	15 wt% H_2O in 1-Propanol	0.4	21.7 ± 0.3
DMAc	pPIM	15 wt% H_2O in EtOH	0.4	27.8 ± 2.6
DMF	pPIM	15 wt% H_2O in DMF	0.4	27.5 ± 0.8
**DMAc**	**pPIM**	**15 wt% H_2O in DMAc**	**0.4**	**39.6 ± 3.4**
DMAc	pPIM	15 wt% H_2O in DMAc	0.2	26.2 ± 2.3
DMAc	pPIM	15 wt% H_2O in DMAc	0.63	23.1 ± 1.6
DMAc	pPIM	15 wt% H_2O in DMAc	0.8	27.3 ± 2.4
-	D521	15 wt% H_2O in 1-Propanol	0.8	15.8 ± 4.4
**-**	**D521**	**40 wt% H_2O in 1-Propanol**	**0.8**	**42.1 ± 1.4**
-	D521	65 wt% H_2O in 1-Propanol	0.8	16.8 ± 2.2

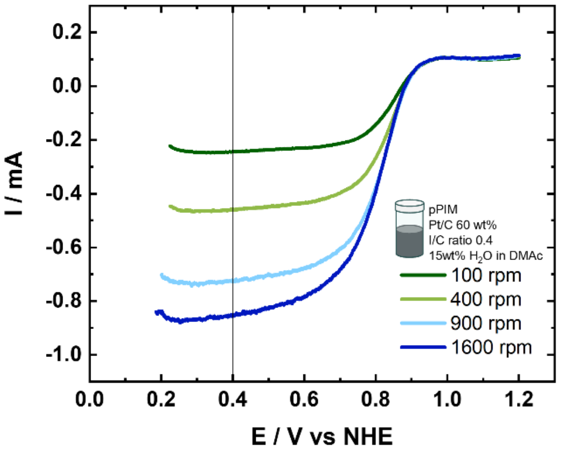

Figure S7. Current-potential curves of a glassy carbon electrode (0.25 cm^2) coated by Pt/C catalyst with a Pt loading of 0.08 mg cm^{-2} using pPIM as ionomer and an I/C ratio of 0.4, recorded at different rotation rates in O_2 saturated 0.5 M H_2SO_4 aqueous solution with a potential scan rate of 20 mV s^{-1}.

Additionally, the pure polymer solution was tested using RDE measurements to obtain the oxygen diffusion coefficient through the polymer material. A 2.5 wt% pPIM polymer solution in DMAc was used. For reference, a D521 dispersion was diluted with water to achieve a 2.5 wt% dispersion. Herein, a platinum electrode was used as the working electrode, a platinum wire was used as the counter electrode, and a normal hydrogen electrode (HydroFlex, Gaskatel) was used as the reference electrode. The working electrode was polished with a 0.05 µm alumina suspension (Allied High Tech production iNC) and sonicated in ultrapure water prior to use. The polymer solutions were pipetted onto the electrode using 10 µL and dried on a heating plate at 60°C. Electrochemical measurements were performed using an ECi-211 potentiostat from Nordic electrochemistry and a rotator with a polyether ether ketone shaft (Pine Research Instrumentation) at room temperature. The electrolyte (0.5 M H_2SO_4) was flushed with high-purity oxygen (O_2) gas for 30 minutes prior to the start of the experiment to ensure a saturated concentration of dissolved O_2. During the measurement, a blanket of O_2 gas was maintained over the surface of the electrolyte to stabilize the concentration of dissolved oxygen. The potential was swept from 0.1 to 1.2 V vs. NHE at 20 mV s^{-1} under a rotating rate of 100 – 1600 rpm. The error stems from the linear regression of the Levich plot. Measuring a 10 µL droplet of each polymer solution on a glass substrate resulted in a thickness of approximately 10 µm.

The oxygen diffusion coefficient for the pPIM thin film is significantly higher (by a factor of 10) than that of the D521 film. This highlights the improved microstructure of pPIM compared to the PFSA reference material.

Table S3. Oxygen diffusion coefficient of thin polymer films measured with RDE.

	**pPIM**	**D521**
D_{O2} / cm^2 s^{-1}	5.66 x10^{-3} ± 1.27 x10^{-3}	5.09 x10^{-4} ± 1.31 x10^{-4}

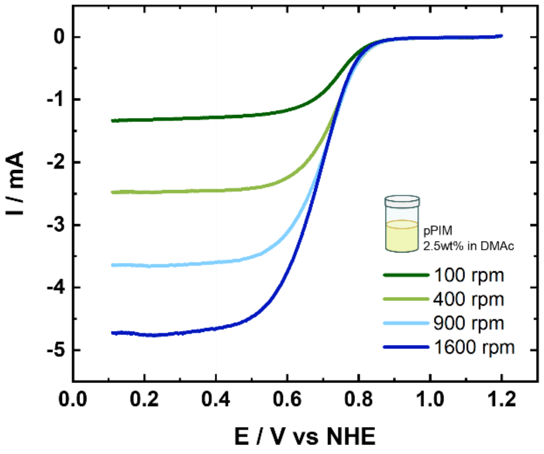

Figure S8. Current-potential curves of a platinum electrode (0.196 cm^2) coated by a pPIM polymer film, recorded at different rotation rates in O_2 saturated 0.5 M H_2SO_4 aqueous solution with a potential scan rate of 20 mV s^{-1}.

Table S4. Anode and cathode Pt loadings of the tested MEAs.

	**pPIM - I/C 0.2**	**pPIM - I/C 0.4**	**pPIM - I/C 0.63**	**pPIM - I/C 0.8**	**D521 - I/C 0.8**
**Ink solvent**	15 wt% H_2O in DMAc	15 wt% H_2O in DMAc	15 wt% H_2O in DMAc	15 wt% H_2O in DMAc	40 wt% in 1-Propanol
**Anode Pt loading / mg cm^{-2}**	0.1333	0.1262	0.1269	0.11843	0.1018
**Cathode Pt loading / mg cm^{-2}**	0.4675	0.4063	0.4339	0.41872	0.4227

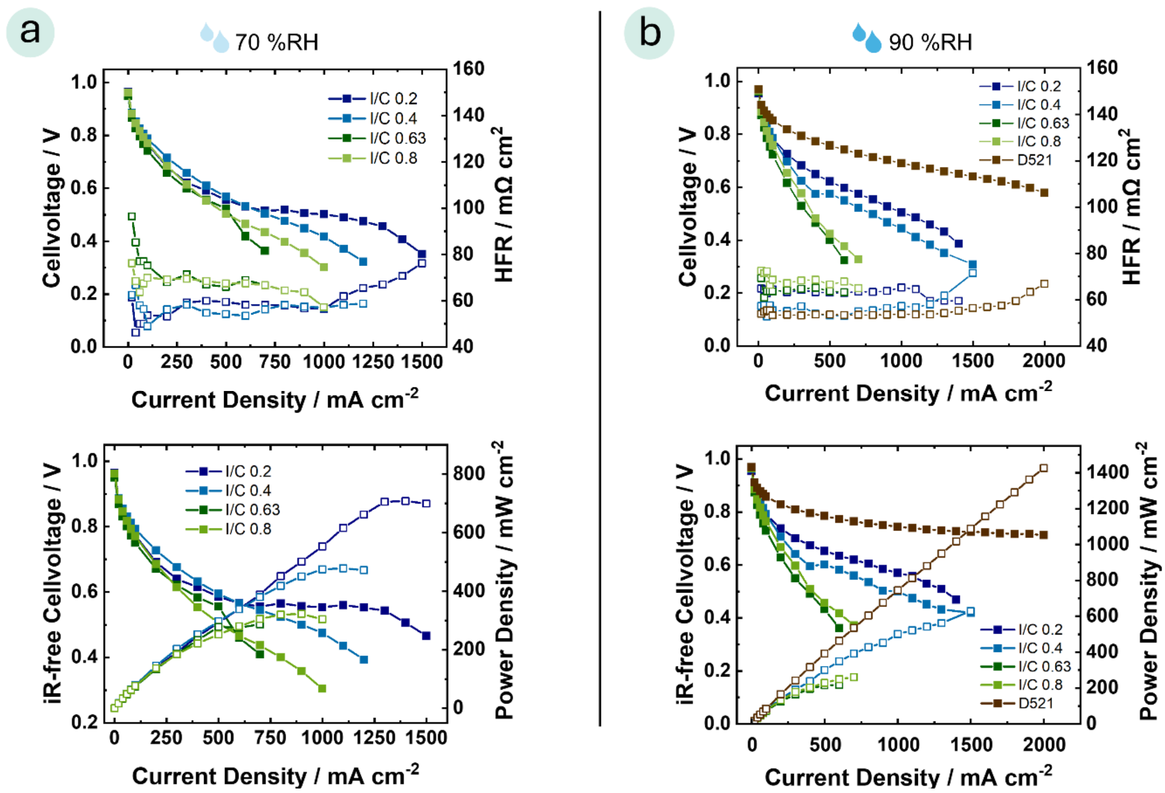

Figure S9. Polarization curves acquired under O_2 with (a) 70 %RH and (b) 90 %RH. (Top) Filled squares correspond to the cell voltage (left y-axis) and open squares correspond to Ohmic resistance (right y-axis). (Bottom) iR-free polarization curves (left y-axis) and power densities depicted with open squares (right y-axis).

Table S5. HFR values /mΩ cm^2 at a current density of 500 mA cm^{-2}.

I/C ratio	70 %RH	90 %RH
0.2	59	63
0.4	54	53
0.63	64	64
0.8	67	67

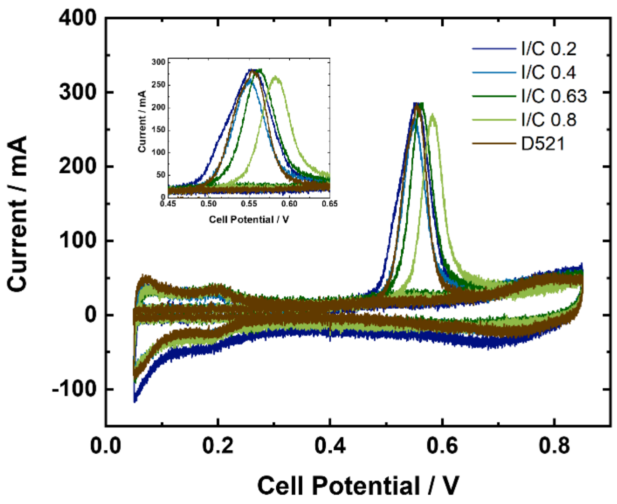

Figure S10. CO-stripping voltammograms for ECSA determination of the cathode catalyst layer using pPIM with different I/C ratios and D521 with an I/C ratio of 0.8 as binder material.

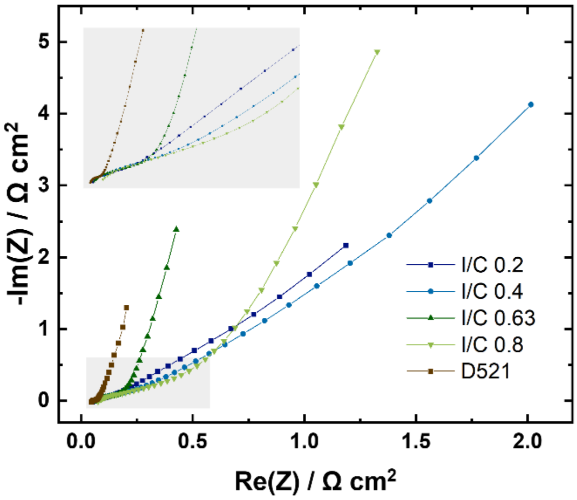

Figure S11. Nyquist polts of EIS when N_2 is flushed at the cathode to determine the protonic sheet resistance using pPIM with different I/C ratios and D521 with an I/C ratio of 0.8 as binder material.

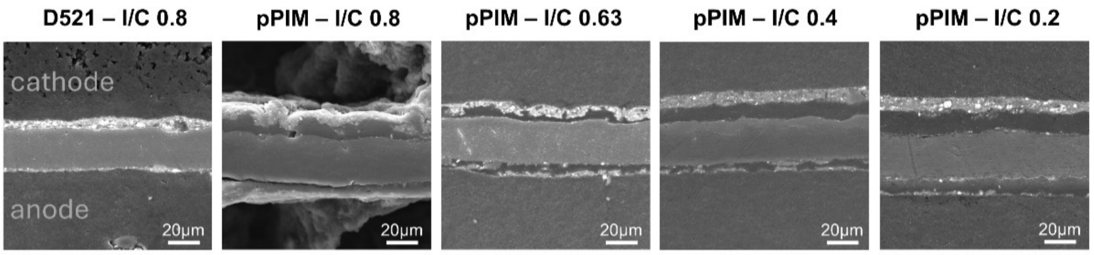

Figure S12. SEM cross-sections of the tested MEAs. Herein, the cathode side is at the top.

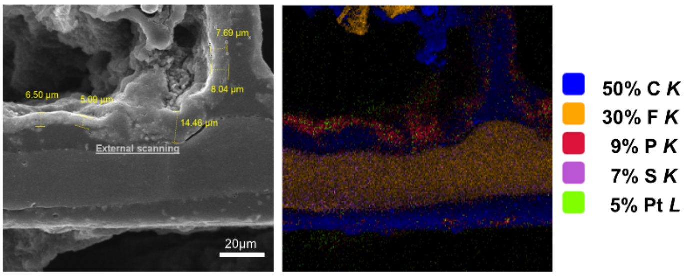

Figure S13. SEM cross-section of the MEA with pPIM using an I/C ratio of 0.8 and corresponding EDX analysis.

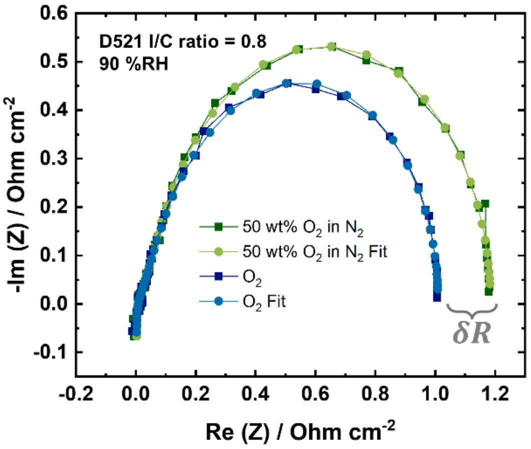

$$D_{oxy} = \frac{(k-1)*b*l_t}{k*8*F*(\delta R)*c_1}$$

k… ratio of oxygen concentrations in two experiments
b… Tafel slope
l_t… catalyst layer thickness
F… Faraday constant
δR… Difference of low frequency CCL resistivities
c_1…oxygen concentration at CCL/GDL interface

Figure S14. Exemplary pEIS at different O_2 concentrations of MEA with D521 as binder in the catalyst layer (FC test parameters: T = 80°C, RH=90%, 2bar-a, under H_2/O_2, cathode loading 0.4 mg cm^{-2}, anode loading 0.1 mg cm^{-2}.
